# Measuring social norms endorsement and normative influence on contraceptive use among youth (15–24) in northeastern Uganda

**DOI:** 10.3389/fgwh.2026.1778239

**Published:** 2026-06-17

**Authors:** Lillian Ojanduru, Justine Bukenya, Dinah Amongin, Elizabeth Ekirapa, Nazarius. M. Tumwesigye, Godfrey Siu

**Affiliations:** 1 School of Public Health, Department of Epidemiology and Biostatistics, Makerere University, Kampala, Uganda; 2 School of Public Health, Department of Community Health and Behavioural Sciences, Makerere University, Kampala, Uganda; 3 School of Public Health, Department of Disease Control and Environmental Health, Makerere University, Kampala, Uganda; 4 School of Public Health, Department of Health Policy, Planning, and Management, Makerere University, Kampala, Uganda; 5 College of Health Sciences, Child Health and Development Centre, Makerere University, Kampala, Uganda

**Keywords:** contraceptives, family planning, sexual and reproductive health, social norms, youth

## Abstract

**Background:**

Despite widespread availability of contraceptives, uptake remains lower than expected given the unmet need. This gap highlights the importance of examining how social norms influence contraceptive use. This study measured young people's endorsement of social and contraceptive use among young people aged 15–24 years in Uganda.

**Methods:**

A cross-sectional study was conducted among 448 youth aged 15–24, of whom 409 were sexually active. A Likert-scale social norms tool, developed by the same research team through literature review and qualitative research, was applied. Eight scales captured domains including provision of sexual education, family planning myths and misconceptions, reproductive health, couple communication, masculinity, sanctions, self-efficacy, and engagement norms. Descriptive statistics summarized participant characteristics and responses, with median scores dichotomized into high vs. low agreement. Associations between social norms and contraceptive use were examined using modified Poisson regression with robust variance estimation, while sensitivity analyses with continuous scores provided predicted probabilities for a nuanced interpretation.

**Results:**

Contraceptive use was strongly predicted by self-efficacy (APR = 3.80, 95% CI: 3.39–4.25) and endorsement of positive reproductive health norms (APR = 4.62, 95% CI: 4.16–5.13), with predicted probabilities rising to near universal levels among high scorers. Rejecting negative masculinity, myths, and restrictive views on sexual education produced moderate gains (APR range 1.12–1.30), while sanctions suppressed uptake (APR = 0.42, 95% CI: 0.16–1.09). Overall, empowerment and supportive norms most strongly promoted contraceptive use, whereas punitive attitudes reduced it

**Conclusion:**

Contraceptive use among youth is most strongly influenced by self-efficacy and positive reproductive health norms, moderately shaped by reductions in restrictive attitudes, and suppressed by sanctions. Interventions that build confidence, promote supportive norms, and address stigma are likely to achieve the greatest gains in uptake.

## 1 Introduction

The role of social norms in shaping sexual and reproductive health (SRH)—particularly sexual behavior, marriage, and pregnancy intentions—has been widely acknowledged by demographers and reproductive health researchers (1). Although earlier scholars noted challenges in defining and measuring social norms (2), subsequent research has provided clearer evidence of their influence on contraceptive use. For instance, Sedlander et al. (3) found that normative beliefs linking contraceptive use to infertility significantly shaped uptake in Kenya (3), while Lahiri et al. (4) documented the role of social norms in adolescent family planning in Kilifi County (4). Conceptual advances from the Learning Collaborative have further refined approaches to understanding and measuring social and gender norms (5). More recently, realist evaluations have shown how norms-shifting interventions foster community-level social and behavior change (6). Thus, while definitional and measurement challenges persist, the growing body of evidence demonstrates that social norms exert a measurable influence on sexual and reproductive health behaviors, and interventions leveraging these dynamics are increasingly effective in sub-Saharan Africa.

In this study, we adopted Cialdini's (7) definition of social norms as unwritten, obligatory rules that prescribe appropriate and acceptable behaviors within a community, enforced by reference groups (7).

In Africa, scholars and practitioners are increasingly integrating social norms strategies to address SRH behaviors among adolescents (8). Contraceptive uptake among adolescents in sub-Saharan Africa remains low, contributing to early pregnancy and abortion (9). Although prevalence varies widely across countries, with some nations achieving relatively high uptake of modern methods while others remain far behind due to cultural, economic, and policy barriers. For instance, Ethiopia (41%), Zimbabwe (66.8%), South Africa (64%) and Kenya (58%) have reported notable improvements in contraceptive use, largely attributed to strong government-led family planning initiatives and community health worker programs (10). In contrast, countries such as Chad and Nigeria continue to struggle with low prevalence rates of 5% and 17% respectively, reflecting persistent challenges in health infrastructure, limited access to services, and sociocultural resistance to modern contraceptive methods (11). These disparities are well documented in recent scholarship, which emphasizes the role of social norms, education, and health system capacity in shaping adolescent contraceptive decision-making (11–13).

In Uganda, modern contraceptive use has risen to 38% among married women aged 15%–49% and 33% among all women (14). However, in Karamoja—a remote pastoral community in Northeastern Uganda—demand for family planning is low (27.3%), with contraceptive uptake at only 10% among married and unmarried women (14) This low uptake is largely attributed to strong cultural influences, social norms, and beliefs that discourage contraceptive use, particularly among young women and adolescents (15). The effect of social norms on contraceptive use is often underestimated, given the complexity of measuring how common, how strong, and how both individual and collective norms (shared expectations, values, and unwritten rules that guide behavior within a group or community) evolve over time (16–18). Collective norms shape how individuals act, what they believe others expect of them, and how society functions at large (19, 20).

In rural sub-Saharan Africa, collective norms around sexuality and fertility emphasize early childbearing, discourage premarital sexual activity, and stigmatize contraceptive use among unmarried adolescents (19, 21). Stigma reinforces these expectations, with contraceptive use often perceived as promiscuity, undermining adolescents' social standing and discouraging service uptake (21). Fear of judgment from providers, families, or peers leads many to avoid health facilities or conceal contraceptive use, resulting in risks such as unprotected sex or reliance on unreliable methods, and adverse outcomes including unintended pregnancy, maternal morbidity, unsafe abortion, and school dropout (22). Evidence confirms that collective fertility and gender norms are linked to lower contraceptive uptake, while stigma and provider bias remain critical barriers to adolescent reproductive health (19, 21, 22).

Social norms are internalized from an early age and strongly influence how adolescents manage their reproductive health choices (23). They shape perceptions of what is acceptable, desirable, and expected within communities, thereby impacting young people's decision-making abilities (24). Whether consciously or unconsciously, social norms influence health-related behaviors, including contraceptive use (1). Consequently, there is growing interest in examining the role of social norms in SRH behaviors, particularly contraceptive uptake (25, 26).

Existing studies assessing the effect of social norms on contraceptive uptake among adolescents in sub-Saharan Africa are predominantly qualitative (27–29) while quantitative evidence remains limited (18). Recent research has introduced measures of contraceptive autonomy (30), reproductive agency (31) and fertility norms (32) Nonetheless, there remains a gap in measuring social norms that influence sexuality and contraceptive demand among youth, particularly in remote pastoral communities in low- and middle-income countries (LMICs). This challenge is compounded by the absence of culturally appropriate quantitative tools. Without such tools, the effect of social norms may be underestimated, thereby diminishing recognition of efforts to address them (33).

## 2 Methodology

### 2.1 Study design and study site

This study employed a cross-sectional design targeting individuals aged 15–24 years, with data collected using quantitative methods. The study was conducted between July 2022 and June 2023 across three districts of the Karamoja region: Nakapiripirit, Napak, and Nabilatuk.

Karamoja is a remote pastoral area characterized by strong cultural traditions, including postpartum abstinence as a means of birth spacing. In this practice, men often relocate to the kraal (livestock enclosures) and remain away from home for two or more years to delay subsequent births. However, this custom is gradually declining due to the increasing adoption of agro-pastoral livelihoods.

The region maintains deeply rooted cultural institutions, notably the Akiriket (Council of Elders), who exert considerable influence in guiding and preserving traditions. A high cultural value is placed on large families, with many children regarded as a source of social and economic strength.

### 2.2 Sample size estimation and sampling

#### 2.2.1 Sample size estimation

Using Taro Yamane's sample size calculation formula (1967), we determined a sample size of 400 youth for this study. To account for study design considerations, we applied a 90% statistical power and incorporated a 10% non-response rate at a precision level of 0.05 (34). This adjustment resulted in a final sample size of 448 participants. A simple proportionate distribution of the sample size was performed to determine the number of respondents per district.

Taro Yamane's formula (1967), was appropriate for determining representative samples from finite populations when the primary objective is to estimate proportions (e.g proportion of youth who endorsed social norms) rather than detect differences between means. In this study, our aim was to assess normative influence on contraceptive use by estimating the prevalence of contraceptive use among youth who endorsed or disagreed with social norms with a specified level of precision (margin of error = 0.05). The application of 90% statistical power was incorporated to ensure robustness of the estimates, particularly because the same sample was also used in a related study where hypothesis testing was required.

$$n=\frac{N}{1+N(e^{2})}\ldots \ldots \ldots \ldots \ldots ..1$$

#### 2.2.2 Sampling

The researchers employed a simple random sampling technique to select both the study sites and participants. All sub-counties within the selected districts were listed on slips of paper and thoroughly mixed (lottery method). A representative from each district was then asked to randomly pick two sub-counties. Within each selected sub-county, all parishes were similarly listed, mixed, and two parishes were randomly chosen. Finally, all villages within the selected parishes were included in the study.

Households were then systematically sampled. At the village level, household lists were obtained from the Local Council one (LC1) offices. In cases where these lists were not updated, research assistants worked together with the local councils to revise and update them prior to sampling. Systematic sampling was then applied: the total number of households in each village was divided by the required sample size from that village to determine the sampling interval. A random starting point was chosen within the first interval, after which every kth household (based on the calculated interval) was selected until the desired sample size was reached. The sample intervals varied from village to village In instances where a selected household contained more than one youth aged 15–24 years, one individual was randomly selected using the lottery method to participate in the interview.

### 2.3 Inclusion and exclusion criteria

Youth aged 15–24 years who had resided in the community for a minimum of 12 months and voluntarily consented to participate were included in the study. Exclusion criteria comprised individuals who did not consent to participate despite being eligible, those who could not participate because they were sick by the time of interviews and those younger than 18 years whose parents or guardians did not provide informed consent.

### 2.4 Data collection methods and tools

This study applied a social norms measurement tool that was rigorously developed by the research team in earlier work through a multi-phase process to ensure reliability and contextual relevance. First, the team conducted a comprehensive review of existing instruments such as the Gender Equitable Men (GEM) scale, Sexual Relations Scale, Fertility Norm Scales, Couple Communications Scale, Gender Norms Scale, and Reproductive Health Scale to identify gaps (35–39). The scales derived from literature included: 1. Provision of sexual education—norms restricting provision of sexual education. 2. Family planning myths and misconceptions—e.g., the belief that women using contraception may become promiscuous. 3. Reproductive health scale—positive norms supporting SRH. 4. Couple communication scale—positive norms promoting communication between partners on contraceptive use. This was followed by a qualitative study to explore prevailing norms within the study population. Insights from both phases informed the creation of a quantitative tool, with social norm scales constructed using exploratory factor analysis (EFA) and polychoric correlation matrices to account for the ordinal nature of Likert-scale responses. Factors were extracted and tested for reliability using Cronbach's alpha and McDonald's Omega coefficients (Ωt and Ωh), applying conventional thresholds (≥.70 acceptable, ≥.80 good, ≥.90 excellent). An Ωh ≥ .50 was interpreted as evidence of a meaningful general factor, ensuring robustness of the scales. The scales developed from the qualitative study included: 5. Gender and masculinity scale—e.g., the belief that joint household decision-making undermines male authority. 6. Sanctions scale—norms reflecting potential punishments for violating rules on sexuality and contraceptive use. 7. Self-efficacy scale—norms related to one's ability to access and use family planning independently (e.g., obtaining a method without permission). 8. Engagement sex and courtship norm scale—norms around culturally acceptable premarital sex as a pathway to marriage. A separate manuscript detailing the measurement approach has been submitted for publication elsewhere; therefore, this paper focuses on the application of the tool rather than its full development process.

### 2.5 Data collection procedures

Data were collected structured questionnaires administered in face-to-face interviews by trained research assistants. Interviews were conducted in participants' preferred language to enhance comprehension and accuracy. Each participant was assigned a unique ID.

Data were collected from Napak, Nabilatuk and Nakapiripirit Districts in Karamoja region, Northeastern Uganda using handheld devices programmed with Kobo collect. Built-in skip patterns and validation checks minimized errors. Supervisors reviewed data daily for completeness and consistency. Data were securely transmitted to a server and backed up on encrypted, password-protected laptops. Data was later downloaded for analysis.

### 2.6 Variable measurement

#### 2.6.1 Dependent/outcome Variable

The primary outcome variable was contraceptive use. Respondents who had engaged in sexual activity within the past 12 months were asked:

“Have you or your partner ever used a contraceptive method?” (coded as 1 = Yes, 2 = No)

“Are you currently using any form of contraceptive method?” (coded as 1 = Yes, 2 = No)

#### 2.6.2 Independent variables

Independent variables were social norms, that were constructed using Exploratory Factor Analysis (EFA). Each social norm scale comprised 3–7 items coded as: 1 = Agree, 2 = Partially agree, 3 = Disagree except for engagement sex (rape) which was a single item. Research participants were asked if they agreed, partially agreed or disagreed with each of the items in the scale. Eight scales included as independent variables were;- provision of sexual education, family planning myths and misconceptions, reproductive health, couple communication, masculinity, sanctions, self-efficacy, and engagement norms.

#### 2.6.3 Data analysis

##### 2.6.3.1 Descriptive statistics

Descriptive statistics were first computed to summarize participant characteristics and the distribution of responses to social norm items. For each domain, summative scores were calculated and median values derived. In the primary analysis, median scores were used to create dichotomous variables indicating high vs. low levels of agreement. A high score for positive norms was considered desirable, as it reflected endorsement, whereas a low score was desirable for negative norms, as it reflected disagreement.

##### 2.6.3.2 Multivariable analysis

Modified Poisson regression with robust variance estimation was employed to estimate adjusted prevalence ratios (APRs) and 95% confidence intervals for the association between social norms and contraceptive use. This approach was chosen over logistic regression because contraceptive use was relatively common (11%); odds ratios can overestimate associations when outcomes exceed 10%. Modified Poisson regression directly estimates APRs, which approximate risk ratios and are more interpretable in cross-sectional studies.

To address potential information loss from dichotomization, sensitivity analyses retained summative scores in continuous form. These were entered into regression models to test consistency across approaches. APRs from continuous models were translated into predicted probabilities of contraceptive use, using observed baseline prevalence within each reference group. This quantified changes in probability associated with incremental shifts in norm endorsement, providing a more nuanced understanding of normative influences. Presenting both dichotomized and continuous measures ensured robustness and interpretability.

### 2.6 Ethical considerations

This study was conducted in accordance with the ethical principles enshrined in the Helsinki Declaration. Ethical approval was obtained from the Makerere University School of Public Health Research Ethics Committee (Approval Number: SPH-2022-294) and the Uganda National Council of Science and Technology (UNCST), the national research regulatory body (Approval Number: HS2547ES). All respondents provided written informed consent prior to participation and were assured of safety, confidentiality, and voluntary participation, including the right to withdraw from the survey at any time without penalty. For participants younger than 18 years, informed consent was obtained from their parents or guardians, while assent was obtained from the minors themselves.

## 3 Results

### 3.1 Socio-demographic characteristics of study participants and contraceptive use

In Table 1, a total of 448 youth participated in the study, of whom 50% were male. The majority (79.5%) were aged 20–24 years. Approximately one-third (37.1%) lived with their biological parents, with a higher proportion among males (43.3%) compared to females (20.8%). More than half (54.2%) had attained primary-level education (53.1% males vs. 55.4% females). Overall, 62.3% were not employed, with unemployment higher among males (71.0%) than females (53.6%). Among those employed, peasant agriculture was the main source of livelihood (16.3%), with male youth more commonly engaged in pastoralism. Nearly 35% reported listening to the radio almost daily, which served as their main source of information.

**Table 1:** Characteristics of young people and modern contraceptive use among sexually active youth.

Background Characteristics	Male (*n* = 224)	Female (*n* = 224)	All (*n* = 448)	*P*-value
	*n* (%)	*n* (%)	*n* (%)	
Age (years)				
15–19	46 (20.5)	46 (20.5)	92 (20.5)	
20–24	178 (79.5)	178 (79.5)	356 (79.5)	
Live with				
Biological parents	97 (43.3)	69 (20.8)	166 (37.1)	
Guardians	27 (12.1)	15 (6.7)	42 (9.4)	***
Older siblings	11 (4.9)	2 (0.9)	13 (2.9)	
Own	86 (38.4)	32 (14.3)	118 (26.3)	
Husband/wife	3 (1.3)	106 (47.3)	109 (24.3)	
Education Level				
No education	54 (24.1)	67 (29.9)	121 (27.0)	
Primary	119 (53.1)	124 (55.4)	243 (54.2)	
Secondary +	51 (22.8)	33 (14.7)	84 (18.8)	
Type of Job				
No Job	159 (71.0)	120 (53.6)	279 (62.3)	
Farming/agriculture	29 (13.0)	44 (19.6)	73 (16.3)	
Formal paid work	4 (1.8)	14 (6.3)	18 (4.0)	***
Informal paid work	32 (14.3)	20 (8.9)	52 (11.6)	
Other	0 (0.0)	26 (11.6)	26 (5.8)	
Religion				
Anglican	45 (20.1)	89 (89.7)	134 (29.9)	
Catholic	167 (74.6)	118 (52.7)	285 (63.6)	***
Moslem	11 (4.9)	12 (5.4)	23 (5.1)	
Other	1 (0.5)	5 (2.2)	6 (1.4)	
Listening to a radio				
Almost everyday	82 (36.6)	75 (33.5)	157 (35.0)	***
Once a week	91 (40.6)	48 (21.4)	139 (31.0)	
Less than once a week	7 (3.1)	29 (13.0)	36 (8.0)	
Not at all	44 (19.6)	72 (32.1)	116 (26.0)	
Ever had sex				
Yes	219 (97.8)	190 (84.8)	409 (91.3)	***
No	5 (2.2)	34 (15.2)	39 (8.7)	
Sexual and reproductive health (*n* = 409)				
Currently in a relationship				
Yes	194 (88.6)	178 (89.7)	372 (91)	
No	25 (11.4)	12 (6.3)	37 (0.9)	
Marital Status				
Married or living together.	98 (44.8)	136 (71.6)	234 (57.2)	
	7 (3.2)	13 (6.8)	20 (4.9)	***
Separated/divorced.	114 (52.0)	41 (21.6)	155 (37.9)	
Never Married				
Have Children				
Yes	94 (42.9)	161 (84.7)	225 (62.4)	
No	125 (27.1)	29 (15.3)	154 (37.6)	
Currently using FP				
Yes	14 (6.4)	31 (16.3)	45 (11.0)	
No	205 (93.6)	159 (83.7)	364 (89.0	**
Ever use of FP				
Yes	108 (49.3)	62 (32.6)	170 (41.6)	
No	97 (44.3)	127 (66.9)	224 (54.8)	***
Don't know	14 (6.4)	1 (0.5)	15 (43.7)	
Contraceptive methods ever used				
Males (*n* = 108) females (*n* = 62) Total (*n* = 170)				
IUD	4 (1.8)	4 (2.1)	8 (1.9)	
Injectables	8 (3.6)	21 (11.1)	29 (7.1)	**
Implants	9 (4.1)	14 (7.4)	23 (5.6)	
Pills	22 (10.0)	2 (1.0)	24 (5.9)	***
Condoms	69 (31.5)	8 (4.2)	77 (18.8)	***
Emergency contraceptives	11 (5.0)	2 (1.0)	13 (3.2)	

*** *p* < 0.001.

** *p* < 0.01.

* *p* < 0.05.

Some people used both condom and another method.

Regarding sexual and reproductive health, 91.3% reported having ever had sex (98% males vs. 84% females). Slightly more than half (57.2%) were married, with a higher proportion among females (71.6%) compared to males (44.8%). Overall, 62% had children, though this was more common among females (84.7%) than males (42.9%). Contraceptive use varied across participants. About 41.6% had ever used some form of contraceptive, with prevalence higher among males (49.3%) than females (32.6%). However, only 11.0% were currently using some form of contraceptives (6.4% males vs. 16.3% females). The most commonly reported method used was male condoms (18.8%), with a significant gender difference (31.5% males vs. 4.2% females; *p* < 0.001). Among females, the most frequently used methods were injectables (11.1%), followed by implants (7.4%) and oral pills (Table 1).

### 3.2 Level of young people's endorsement of social norms and contraceptive use

In Table 2, bivariate analysis was conducted to assess gender differences in agreement with social norms related to contraceptive use and sexual and reproductive health (SRH). Results presented in Table 3 show that overall, agreement with social norms varied significantly by gender, with females generally more supportive than males. For example, 62.0% of females compared to 25.5% of males agreed with norms prohibiting the provision of sexual education (*p* < 0.001). Similarly, 57.2% of females and 19.7% of males agreed that unmarried youth should not receive SRH education, including contraceptives (*p* < 0.001). Acceptance of engagement sex—a cultural practice in which a boy is assisted by peers to forcefully have sex with a girl so she becomes his wife—was high overall (51.6%), but significantly higher among females (77.2%) than males (25.9%) (*p* < 0.001). Regarding negative masculinity norms, males were significantly more likely than females to agree that contraceptives are a woman's responsibility or that women discussing contraceptives undermine masculinity (*p* < 0.001). Conversely, females were more supportive of reproductive health norms, such as birth control being part of responsible sexuality and men sharing responsibility for contraception (*p* < 0.001).

**Table 2:** Agreement with social norms related to contraceptive Use and SRH by gender.

Statement	Female (*n* = 224)	Male (*n* = 224)	All (*n* = 448)	*p*-value
	*n* (%)	*n* (%)	*n* (%)	
Provision of Sexual Education				
Unmarried youth do not need to receive education on SRH including contraceptives.	128 (57.1)	44 (19.6)	172 (38.4)	***
Educating youth about preventing pregnancies leads to sexual immorality	167 (74.6)	91 (40.6)	258 (57.6)	***
The best way to prevent unintended pregnancies among young people is to keep them in the dark	115 (51.3)	51 (22.8)	166 (37.1)	***
Overall agreement	139 (62.1)	57 (25.4)	196 (43.8)	***
Engagement sex norm				
A boy who is interested in a girl for wife can organize and rape the girl without considering that as a crime	173 (77.2)	58 (25.9)	231 (51.6)	***
Myths & Misconceptions				
Woman using contraception may become promiscuous	119 (53.1)	121 (54.0)	240 (53.6)	ns
If a youth who has not given birth uses contraceptives, s/he will become infertile	153 (68.3)	111 (49.6)	264 (58.9)	***
Contraception causes cancer	126 (56.3)	154 (68.8)	280 (62.5)	**
You will give birth to deformed baby if you use contraceptives	112 (50.0)	131 (58.5)	243 (54.3)	***
Overall agreement	116 (51.8)	129 (57.6)	245 (54.7)	ns
Gender and Masculinity				
A woman who decides in her household together with a man makes the man feel less of a man	78 (34.8)	144 (64.3)	222 (49.6)	***
It is not acceptable for a woman to bring up the topic of FP with her husband	70 (31.3)	39 (17.4)	109 (24.3)	***
A man should not discuss contraception with his wife	54 (24.1)	22 (9.8)	76 (17.0)	***
Contraception is a woman's business, and a man should not have to worry about it	49 (21.9)	49 (21.9)	98 (21.9)	***
A man should have the final decisions on when and how many children to have	57 (25.4)	123 (54.9)	180 (40.2)	***
Overall agreement	61 (27.2)	76 (33.9)	137 (30.6)	***
Sanctions				
If I use FP, I will be ridiculed by my peers	15 (6.7)	43 (19.2)	58 (12.9)	***
If I use FP, I will be punished by my family	18 (8.0)	25 (11.2)	43 (9.6)	***
If I use FP, the council of elders will punish me	1 (0.4)	3 (1.3)	4 (0.9)	ns
Fellow youth will beat me during “Ametho”- Organized public beating	0 (0.0)	2 (0.9)	2 (0.5)	ns
If a married women is raped, the perpetrator must pay cows equivalent to the number that was paid as dowry	126 (56.3)	185 (82.6)	311 (69.4)	***
Overall agreement	110 (49.1)	91 (40.6)	201 (44.9)	ns
Reproductive Health Norms				
Birth control is part of responsible sexuality	189 (84.4)	162 (72.3)	351 (78.4)	***
A man should share responsibility for birth control	178 (79.5)	161 (71.9)	339 (75.7)	**
Couples in my community should use FP if they want to prevent pregnancy	200 (89.3)	120 (53.6)	320 (71.4)	***
Overall agreement	218 (97.3)	145 (64.7)	363 (81.0)	***
Couple Communication				
Husbands and wives should talk about when to have children.	174 (77.7)	197 (88.0)	371 (82.8)	**
A woman should first discuss family planning use with her husband before taking a method	133 (59.4)	172 (76.8)	305 (68.1)	***
Couples who decide together to use FP methods have better relationships than couples who do not.	155 (69.2)	210 (93.8)	365 (81.5)	***
Overall agreement	187 (83.5)	210 (93.8)	397 (88.6)	**
Self-Efficacy				
I have intention to use FP in the next 6 months	70 (31.3)	56 (25.0)	126 (28.1)	ns
I know where to get family planning methods	184 (82.1)	156 (69.6)	340 (75.9)	**
I would feel comfortable getting FP if I wanted	187 (83.5)	100 (44.6)	287 (64.1)	***
I can get FP method anytime if you wanted to without getting permission from another person?	172 (76.8)	100 (44.6)	272 (60.7)	***
I have control over resources that I could use to access FP	190 (84.8)	87 (38.8)	277 (61.8)	***
Overall agreement	57 (25.4)	120 (53.6)	177 (39.5)	***

*** *p* < 0.001.

** *p* < 0.01.

* *p* < 0.05.

ns, not significant.

**Table 3:** Associations between social norms and contraceptive Use: regression results.

**Variable (Reference Group)**	**Ever Use (%)**	**Never Use (%)**	**Adjusted APR (95% CI)**	**Baseline Probability**	**New Probability**	**Change vs. Baseline**	***P*-Value**
Sexual education (low ≤8)	25.7	74.3	1.12 (1.01–1.21)	25.7%	28.8%	+3.1%	**
Myths and Misconceptions (high myths)	29.9	70.1	1.15 (1.03–1.28)	29.9%	34.4%	+4.5%	**
Engagement norms (low ≤5)	35.0	65.0	1.35 (1.11–1.35)	35.0%	47.3%	+12.3%	*
Negative gender and masculinity (high >8)	27.8	72.2	1.30 (1.24–1.51)	27.8%	36.1%	+8.3%	***
Sanctions (high >8)	38.1	61.4	0.42 (0.16–1.09)	38.1%	16.0%	–22.1%	
Reproductive health norms (low ≤6)	22.3	77.6	3.85 (1.89–7.83)	22.3%	85.8%	+63.5%	***
Couple communication (low ≤ median)	15.7	84.3	1.14 (0.98–1.35)	15.7%	17.9%	+2.2%	***
Self-efficacy (low ≤8)	45.0	55.0	3.80 (3.39–4.25)	45.0%	100%	+55.0%	***

*** *p* < 0.001.

** *p* < 0.01.

* *p* < 0.05.

For couple communication norms, males were significantly more likely to agree compared to females (*p* < 0.01). No significant gender differences were observed in relation to contraceptive myths/misconceptions or sanctions. Finally, males reported significantly higher self-efficacy to access and use contraceptives compared to females (*p* < 0.001).

### 3.3 Associations between social norms and contraceptive use

Table 3 summarizes associations between social norms and contraceptive use among young people. When engagement sex was analyzed separately from other courtship norms, rejecting this practice was associated with a notable 12% increase in contraceptive use, underscoring its independent influence despite being measured as a single item (APR = 1.12, 95% CI: 1.01–1.21), *p* < 0.05, while reduced agreement with myths and misconceptions corresponded to a 15% increase in contraceptive use (APR = 1.15, 95% CI: 1.03–1.28), *p* < 0.01. Rejecting negative gender and masculinity norms was associated with a 30% higher prevalence (APR = 1.30, 95% CI: 1.24–1.51), *p* < 0.001. Support for couple communication produced a modest gain (APR = 1.14, 95% CI: 0.98–1.35), *p* < 0.001.

Positive reproductive health norms and self-efficacy were the strongest predictors. Endorsement of reproductive health norms significantly increased contraceptive use more than fourfold (APR = 4.62, 95% CI: 4.16–5.13) *p* < 0.001, while high self-efficacy nearly quadrupled prevalence (APR = 3.80, 95% CI: 3.39–4.25) *p* < 0.001. Translating APRs into predicted probabilities highlighted the magnitude of these effects: contraceptive use rose from 22% to 86% with reproductive health endorsement, and from 45% to nearly universal use among those with high self-efficacy.

Sanctions exerted a suppressive influence. Youth reporting high sanctions had a baseline prevalence of 38%, but the adjusted APR of 0.42 (95% CI: 0.16–1.09) *P* > 0.05 reduced predicted probability to 16%, underscoring the impact of punitive or stigmatizing attitudes. Moderate gains were observed for sexual education, myths and misconceptions, and couple communication, each contributing incremental increases of 2–5 percentage points.

Overall, contraceptive use was most sensitive to improvements in self-efficacy and reproductive health norms, moderately responsive to reductions in negative masculinity, and negatively affected by sanctions. Educational and attitudinal factors contributed to smaller but significant gains. These findings suggest that interventions prioritizing empowerment and normative change, while addressing stigma and sanctions, are likely to yield the greatest improvements in contraceptive uptake.

## 4 Discussion

The findings suggest that reduced agreement with negative social norms plays a critical role in promoting contraceptive use among young people. Youth who did not endorse norms prohibiting the provision of sexual education demonstrated a 12% higher prevalence of contraceptive use, underscoring the importance of comprehensive sexuality education in shaping positive reproductive health outcomes. Sexual education equips young people with the knowledge, skills, attitudes, and values necessary to protect their health, develop respectful social and sexual relationships, make responsible choices, understand and safeguard the rights of others (40).

In Uganda, the National Sexuality Education Framework (2018) defines sexuality education as a lifelong process of acquiring information and forming attitudes, beliefs, and values about vital issues such as sexual development, reproductive health, interpersonal relationships, affection, intimacy, body image, and gender roles (41). Evidence indicates that sexual education has positive effects, including increased knowledge and improved attitudes related to sexual and reproductive health and behaviors (42). High-quality sexual education consistently delivers positive health outcomes with lifelong impacts (43). Research further shows that young people are more likely to delay the onset of sexual activity—and, when they do engage in sexual relations, to practice safer sex—when they are better informed about their sexuality, sexual health, and rights (44).

Similarly, rejecting engagement sex norms increased contraceptive use by +12.3%, showing a meaningful effect even as a single item, highlighting how challenging entrenched cultural practices can directly influence reproductive health behaviors. Engagement sex, a form of culturally accepted premarital sex intended to secure a wife in the Karamoja region, reflects deeply rooted traditions that shape sexual and reproductive practices. Previous studies have shown that social norms discouraging premarital sex and contraceptive use are linked to lower contraceptive uptake (1, 33, 45). Consistent with this evidence, our study found that contraceptive prevalence was higher among youth who rejected harmful and coercive forms of engagement sex.

Evidence from several African countries indicates that adolescent girls are often forced into early marriage to preserve virginity and avoid childbirth outside of marriage (14). In many settings, premarital sex is stigmatized, and sexual activity is considered acceptable only within marriage, contributing to low contraceptive use among unmarried youth (46) A meta-analysis by Wang (47) estimated the global prevalence of premarital sexual intercourse at 41.9% (95% CI: 34.2%–49.6%), with a pooled contraceptive prevalence rate (CPR) of 57.0% (95% CI: 44.3%–69.8%) overall and 57.6% (95% CI: 39.5%–75.6%) at first intercourse (47). In Karamoja, engagement sex is practiced as a pathway to marriage, with the expectation that the wife will bear children (48). Such unprotected premarital sex may be driven not only by cultural traditions but also by limited access to sexual and reproductive health education (49).

The results demonstrate that challenging negative social norms is central to improving contraceptive uptake among young people. Youth who rejected myths and misconceptions about contraception reported a 15% higher prevalence of use. Misconceptions often arise from limited access to family planning information, as inadequate communication fosters rumors, incorrect beliefs about contraceptive methods, and exaggerated concerns about side effects (50, 51). Service delivery challenges also contribute to misinformation: health workers frequently rush through counseling, leaving clients dissatisfied, while heavy workloads in already overstretched facilities limit the time available for detailed guidance (52). In Karamoja, for example, the health system is severely under-resourced, with 126 health centers—most of which are HC IIs—supported by only one regional referral hospital and four general hospitals. Staffing levels remain far below WHO targets, with approximately 20,000 people per health unit, 50,000 people per doctor, and 16,882 people per midwife or nurse (53).

In Uganda, fears of side effects remain a leading reason for non-use or discontinuation of contraceptives (14). Similar patterns have been observed elsewhere: in South Africa, rumors, myths, and sociocultural norms significantly hinder contraceptive uptake among adolescent girls and young women (51). Multivariate analyses in Kenya, Nigeria, and Senegal confirm a negative association between belief in myths and contraceptive use across diverse contexts (54). In many settings, misconceptions persist that family planning promotes promiscuity or causes sterility (55, 56). In Karamoja, cultural perceptions further reinforce non-use, with some women believing that birth spacing should occur naturally and expressing fears that contraceptives might “block the uterus” (48).

The findings also highlight the critical role of interpersonal dynamics in contraceptive uptake. Youth who supported couple communication reported a 14% higher prevalence of contraceptive use compared to those who did not, suggesting that fostering open dialogue between partners can strengthen joint decision-making and increase adoption. Evidence from multiple studies confirms this association: couples' communication has been linked to greater uptake of modern contraception among both men (e.g., vasectomy) and women (57–59). In Senegal, couples in which one partner reported discussing family planning were 25 percentage points more likely to use contraception than those who did not, while couples where both partners reported discussion had a 56-percentage point higher likelihood of use—more than twice the effect size (60). Similarly, a study of young couples in Niger found that those who discussed contraceptive use were more likely to report use overtly rather than covertly (61). Other research further supports the positive correlation between couple communication and contraceptive uptake (62, 63).

Consistent with this evidence, our study found that contraceptive use was higher among youth who strongly agreed with the importance of couple communication. However, communication between partners can be hindered by several factors, including peer influence, cultural taboos, and religious beliefs that discourage open discussion about sex among young people (64). Addressing these barriers is therefore essential to strengthening couple communication and improving contraceptive use.

Reduced agreement with negative masculinity norms was associated with a 30% higher prevalence of contraceptive use, underscoring the importance of addressing gendered expectations and promoting equitable attitudes in reproductive health programming. Masculine norms related to decision-making, contraceptive use, and social status—often reinforced by fears of losing power—can make men reluctant to engage in family planning (18). In our study, masculine norms were measured in relation to decision-making on contraceptive use, and results showed that lower agreement with these negative norms was linked to higher contraceptive uptake. Evidence from other studies supports this finding. Limited decision-making power among women has been consistently associated with lower contraceptive use (65) Male partners may also negatively influence family planning by obstructing contraceptive use, leading to discontinuation or covert use (66). In some cases, men are uncertain about their role in contraceptive decision-making (67), while concerns about side effects further shape their attitudes and choices (68). These findings highlight the need for gender-transformative approaches that engage men, clarify their roles, and address misconceptions to foster supportive environments for contraceptive use.

While our study did not find significant relationships between sanctions and contraceptive use, previous research indicates that sanctions related to family planning can reduce uptake (18). In Karamoja, low contraceptive use has been attributed to socio-cultural factors such as the belief that large families confer status and pride, limited access to services, misconceptions, and lack of male support (69). Young people are also influenced by peer and parental disapproval of contraceptive use, with evidence showing that negative attitudes from friends, parents, and partners—often rooted in rumors and perceived side effects—impede adolescent girls' and young women's access to and use of contraceptives (51).

Evidence from other contexts highlights the importance of self-efficacy in contraceptive uptake. A study in Kenya and Nigeria found that high self-efficacy was associated with modern contraceptive use at 12 months postpartum (70). Similarly, qualitative research in Mozambique showed that fostering protective factors such as hope and self-efficacy among young women helped prevent pregnancy and child marriage (71). Hamidi (72) further reported that prescription contraceptive use was significantly higher among individuals with high self-efficacy (AOR = 1.75; 95% CI: 1.29–2.37). Consistent with these findings, our study demonstrated that higher self-efficacy was associated with greater contraceptive use, whereas lower self-efficacy was linked to reduced uptake (72).

## 5 Strengths and limitations

A key strength of this study lies in its use of a social norms measurement tool developed through both literature review and qualitative inquiry, ensuring cultural relevance and validity. Secondly, by focusing on youth aged 15–24, the study addresses a population with high unmet need for contraception, thereby contributing important insights for program design. The research identifies differences between men and women, which is crucial for tailoring interventions. Lastly findings provide actionable evidence for interventions that prioritize sexual education, myth reduction, and male involvement in family planning.

There were some limitations. Self-reported contraceptive use may be subject to social desirability bias, given the sensitivity of sexual health topics in Karamoja region. The study was conducted in Uganda, and findings may not be generalizable to other cultural or geographic contexts. Although the sample size was adequate (448), it may not fully capture the diversity of youth experiences across rural and urban settings.

## 6 Conclusions and recommendations

This study demonstrates that endorsement of negative social norms and myths significantly reduces contraceptive uptake among youth in Karamoja, while strong agreement with positive reproductive health norms and higher self-efficacy markedly increase use. Gender differences were evident, with women more likely to endorse restrictive norms, underscoring the need for gender-sensitive approaches.

Interventions should prioritize the promotion of supportive reproductive health norms, as these were the strongest predictors of contraceptive use. Community dialogues, peer education, and campaigns that frame birth control as part of responsible sexuality and emphasize shared responsibility between men and women are essential. Equally critical is the enhancement of youth self-efficacy through youth-friendly services, counseling, and skills-building initiatives that empower young people to make informed choices.

Programs must also challenge negative masculinity norms that place contraceptive responsibility solely on women or stigmatize female agency. Approaches such as SASA!, REAL Fathers, Engaging Men and Boys, and IRC's EMAP can foster equitable decision-making by promoting open dialogue, debunking myths, and encouraging critical reflection on gendered expectations. Addressing harmful cultural practices, including engagement sex, requires community sensitization, protective policies, and school-based awareness campaigns to safeguard young women.

Reducing sanctions and stigma is equally important, as punitive attitudes were shown to suppress contraceptive use dramatically. Interventions should involve community and faith leaders in reframing contraceptive use as responsible and beneficial, while advocating for the removal of punitive measures. Governments should strengthen policies mandating comprehensive sexuality education, complemented by community-based programs led by NGOs and youth organizations. These initiatives must actively engage women, parents, religious leaders, and cultural leaders to dismantle harmful traditions and create supportive environments for young people's reproductive health choices. Promoting couple communication through relationship workshops and counseling services can also yield meaningful gains.

In conclusion, a multi-pronged strategy is required: empowering youth through self-efficacy, shifting community norms toward reproductive health and gender equity, dismantling harmful practices and sanctions, and expanding education and communication support. By institutionalizing supportive reproductive health norms and creating enabling environments, governments and development partners can significantly increase contraceptive prevalence and improve overall sexual and reproductive health outcomes among young people.

The study underscores the powerful influence of social norms and self-efficacy on contraceptive uptake among youth in Karamoja. The evidence suggests that interventions must go beyond service provision to actively reshape community attitudes and empower young people. Policies that institutionalize comprehensive sexuality education, challenge harmful cultural practices, and promote gender equity are critical. Engaging men and boys in reproductive health conversations, reducing stigma, and dismantling punitive sanctions can create enabling environments for contraceptive use. Development partners and governments should integrate normative change strategies into SRH programs, recognizing that empowerment and supportive norms are stronger drivers of uptake than knowledge alone.

Building on these findings, several areas warrant deeper investigation. First, longitudinal studies are needed to assess how shifts in social norms translate into sustained contraceptive use over time, providing evidence of long-term impact. Second, comparative research across different regions of Uganda and similar cultural contexts would help determine whether the observed patterns are unique to Karamoja or broadly applicable, thereby strengthening generalizability. Third, a gender-focused inquiry into male perspectives and barriers is essential to better understand men's roles and challenges in contraceptive uptake, complementing the current emphasis on female attitudes. Finally, qualitative studies should explore why females are more likely to endorse restrictive norms, offering nuanced insights into cultural, social, and structural drivers, and guiding the design of interventions tailored to gender-specific barriers.

## Data Availability

The raw data supporting the conclusions of this article will be made available by the authors, without undue reservation.
